# QOMIC: quantum optimization for motif identification

**DOI:** 10.1093/bioadv/vbae208

**Published:** 2024-12-24

**Authors:** Hoang M Ngo, Tamim Khatib, My T Thai, Tamer Kahveci

**Affiliations:** Department of Computer and Information Science and Engineering, University of Florida, Gainesville, FL 32611, United States; Department of Computer and Information Science and Engineering, University of Florida, Gainesville, FL 32611, United States; Department of Computer and Information Science and Engineering, University of Florida, Gainesville, FL 32611, United States; Department of Computer and Information Science and Engineering, University of Florida, Gainesville, FL 32611, United States

## Abstract

**Motivation:**

Network motif identification (MI) problem aims to find topological patterns in biological networks. Identifying disjoint motifs is a computationally challenging problem using classical computers. Quantum computers enable solving high complexity problems which do not scale using classical computers. In this article, we develop the first quantum solution, called QOMIC (Quantum Optimization for Motif IdentifiCation), to the MI problem. QOMIC transforms the MI problem using a integer model, which serves as the foundation to develop our quantum solution. We develop and implement the quantum circuit to find motif locations in the given network using this model.

**Results:**

Our experiments demonstrate that QOMIC outperforms the existing solutions developed for the classical computer, in term of motif counts. We also observe that QOMIC can efficiently find motifs in human regulatory networks associated with five neurodegenerative diseases: Alzheimer’s, Parkinson’s, Huntington’s, Amyotrophic Lateral Sclerosis, and Motor Neurone Disease.

**Availability and implementation:**

Our implementation can be found in https://github.com/ngominhhoang/Quantum-Motif-Identification.git.

## 1 Introduction

Biological systems are represented as intricate networks of molecules, such as genes, proteins, and metabolites interacting with each other (Zhu *et al.* 2007). Network motifs are patterns of local interconnections, which occur substantially more frequently in a given network than in a random network of same size (Milo *et al.* 2002). These structures are considered to be the basic building blocks of network topologies, as they provide higher order description of complex biological functions. Motif topologies characterize the structure of networks and explain highly conserved functions saved through interactions among molecules. Motifs have been successfully used in many applications, such as studying the biological processes that regulate transcription (Shen-Orr *et al.* 2002, Alon 2007) or protein interaction networks (Wuchty *et al.* 2003, Ferrè *et al.* 2015), finding important genes that affect the spread of infectious diseases (Wang *et al.* 2014) and revealing relationship across species (Green and Karp 2004, Hu *et al.* 2011).

Motif identification (MI) remains to be a challenging problem as it involves solving the subgraph isomorphism, which is an NP-hard problem (Cook 1971). As the volume of biological data continues to grow rapidly, it will be more computationally expensive to explore motifs in biological networks. Enforcing specific regulatory constraints on interactions, such as activation or repression patterns on the motif topology, further increases the computation time needed to find motifs.

In the literature, there are three well-known measures for counting motifs, namely $F_{1}$, $F_{2}$, and $F_{3}$ (Schreiber and Schwöbbermeyer 2005). $F_{1}$ measures counts every isomorphic subgraph of a target network to a given motif pattern with no restrictions (Kashtan *et al.* 2004, Chen *et al.* 2006, Wernicke 2006, Grochow and Kellis 2007, Kashani *et al.* 2009, Omidi *et al.* 2009). Although $F_{1}$ provides a comprehensive view on all possible embeddings of motif patterns on networks, it fails to capture dependencies among motif embeddings. In addition, $F_{1}$ does not satisfy the downward closure property (Schreiber and Schwöbbermeyer 2005), which can lead to challenges on scaling the motif size. In contrast to $F_{1}$, $F_{2}$, and $F_{3}$ measures impose restrictions on the motif embeddings. Specifically, $F_{2}$ does not allow resulting embeddings of given motifs in the network sharing the same edge (i.e. interaction). $F_{3}$ restricts them from sharing the same node (i.e. molecule). By doing so, $F_{2}$ and $F_{3}$ measures ensure downward closure (Elhesha and Kahveci 2016).

These distinct counting concepts provide various perspectives and trade-offs for the MI problem. For the $F_{2}$ measure, the authors in Patra and Mohapatra (2019a) uses the idea of dynamic expansion trees to count motif embeddings. They use basic building patterns to find embeddings, and then iteratively join the parent patterns with these basic building patterns, which they called pattern-join (Patra and Mohapatra 2019b). In the work (Elhesha and Kahveci 2016), the authors introduced a motif-centric approach which constructs a set of basic building patterns and then explores embeddings corresponding to these patterns in term of $F_{2}$, and $F_{3}$. In the work (Ren *et al.* 2019), the authors consider $F_{3}$ for the MI problem in multi-layer networks. However, other than the topological constraints of $F_{2}$ and $F_{3}$, existing methods for the MI problem do not consider biological constraints within networks, such as the regulatory relations. These are additional constraints that go beyond the motif topology and enforce the type of interactions (such as activation or suppression) for each interaction in the motif. Due to the bottleneck on the current computational capacity, handling the MIs with multiple constraints is challenging. Therefore, a more powerful computing scheme to broaden the scope of this problem is direly needed.

The field of quantum computing has drawn significant attention and investment recently because of its potential supremacy over classical computational methods (Harrow and Montanaro 2017). Specifically, quantum computing can address a variety of tasks which are intractable for classical computers (Shor 1994, Aaronson and Arkhipov 2011, Farhi and Harrow 2019, Cerezo *et al.* 2021). In the field of computational biology, quantum computing with its advantages shows great promise in solving complex computational biology tasks that require substantial computational resources, such as DNA alignment (Sarkar and *et al.* 2021), genome assembly (Boev *et al.* 2021, Charkiewicz and Nowak 2022), and DNA sequence reconstruction (Sarkar *et al.* 2021) (see Marchetti *et al.* 2022 for a short survey).

*Contributions*: In this work, we consider the MI problem, which finds the maximum set of motif embeddings in a target network such that these embeddings do not share any molecule and all of these motif embeddings satisfy the regulatory constraints imposed by the given motif. We refer to our problem as the MI problem. We design a novel quantum solution for the MI problem, namely *QOMIC (Quantum Optimization for Motif IdentifiCation)*. This is the first study solving this generalized MI problem using quantum computing. First, we model the MI problem as an optimization problem on the set of edges of the target network. Then, we propose an integer representation based on this model, followed by the unconstrained objective function for the MI problem. Finally, we introduce a quantum circuit design for solving the MI problem by quantum approximate optimization. We implement QOMIC in the quantum gate-based machine provided by IBM. We compare QOMIC against the baseline methods designed for the classical computer (Milo *et al.* 2002, Paredes and Ribeiro 2015, Ren *et al.* 2019) on 1500 synthetic networks. Our results demonstrate that QOMIC outperforms existing methods in terms of motif count. Our results on real transcriptional regulatory networks for five neurodegenerative disorders suggest that QOMIC efficiently scales to large real networks.

## 2 Methods

In this section, we first provide preliminaries the basic concepts of quantum computing needed for the rest of this article. We then define the MI problem for biological networks. Next, we describe the integer representation of this problem. Finally, we present the construction of the final Hamiltonian and the design of the corresponding quantum circuit.

### 2.1 Preliminaries

Below, we first present basic concepts in quantum computing. Then, we explain the fundamental principles of Quantum Approximate Optimization Algorithm (QAOA), which is needed to understand our quantum computing solution to the MI problem.

*Basic concepts*: At the heart of quantum computing are *quantum bits* (a.k.a. qubits), the quantum analogs of classical bits (0s and 1s). Unlike classical bits, which are either 0 or 1, a qubit can represent 0 and 1 simultaneously, exploiting the principles of quantum superposition. Information stored in a qubit is called the quantum state of that qubit, denoted by |ψ〉. Given two complex numbers $\alpha_{0}$ and $\alpha_{1}$, the quantum state of a qubit can be represented by a linear combination of two basis states |0〉 and |1〉 as:

$$|\psi \rangle =\alpha_{0}|0\rangle +\alpha_{1}|1\rangle$$

Here, $\alpha_{0}$ and $\alpha_{1}$ represent the amplitudes associated with these basic states.

The concept of quantum entanglement allows us to combine multiple qubits into a quantum system, creating a quantum state that encompasses all possible combinations of the individual qubit states. For a system which includes n qubits, the quantum states of the system are presented by $2^{n}$ basic states. For example, given four complex amplitudes $\alpha_{00}$, $\alpha_{01}$, $\alpha_{10}$, and $\alpha_{11}$, a quantum state in a two-qubit system can be represented as:

$$|\Psi \rangle =\alpha_{00}|00\rangle +\alpha_{01}|01\rangle +\alpha_{10}|10\rangle +\alpha_{11}|11\rangle$$

The quantum states of a quantum system can be transformed by *quantum operators*. In the context of quantum gate-based model, these operators are referred to as *quantum gates*. Common gates applied to single qubits include the Pauli-X, Y, Z gates which perform phase flips, and the Hadamard gate which creates superposition by transforming a |0〉 state into an equal superposition of |0〉 and |1〉. Quantum computing also involves gates applied to multiple qubits. One common two-qubit gate is the Controlled NOT (CNOT) gate, which is to flip the state of the second qubit if the first qubit is in the |1〉 state. Additionally, a series of quantum gates applied to qubits is called a *quantum circuit*.

By a process called *measurement*, we extract information from a quantum system. Measurement collapses the qubits’ superposition state into a definite classical state. The outcome of a measurement is probabilistic, because it depends on the qubit’s superposition amplitudes. For example, in a single qubit system with the state of ψ as above, $|\alpha_{0}{|}^{2}$ and $|\alpha_{1}{|}^{2}$ are the probabilities of measuring that system as 0 and 1, respectively.

*Quantum approximate optimization algorithm*: One of the most popular paradigms of quantum computing is the gate-based quantum model (a.k.a. the universal model) (Lloyd 1996). To handle combinatorial optimization problems, a quantum algorithm is introduced to work on the gate-based quantum model, named QAOA (Farhi *et al.* 2014). QAOA is a four-step quantum computing paradigm designed to tackle combinatorial optimization problems.

*Step 1*: Given a combinatorial optimization problem with n binary variables, we define an unconstrained objective function f that quantifies the quality of the solution $S\in {\left\{0,1\right\}}^{n}$ for that problem.

*Step 2*: We construct two quantum operators: *problem Hamiltonian*, denoted as $H_{P}$ and the *mixing Hamiltonian*, denoted as $H_{B}$. Hamiltonian operators govern how the quantum state changes over time through the Schrödinger equation. Specifically, $H_{P}$ is used to encode the objective function of the problem. For any quantum basis state |S〉 corresponding to the solution $S\in {\left\{0,1\right\}}^{n}$, the problem Hamiltonian satisfies $H_{P}|S\rangle =f(S)|S\rangle$. On the other hand, the mixing Hamiltonian $H_{B}$ is used to perform state mixing, facilitating the exploration of solution space. Given $X_{i}$ as the Pauli-X gate that acts on the i*th* qubit, $H_{B}$ can be written as $H_{B}=\sum_{i=1}^{n}X_{i}$.

*Step 3*: Given 2*p* parameters $\left(\gamma ,\beta )\equiv (\gamma_{1},\ldots ,\gamma_{p},\beta_{1},\ldots ,\beta_{p}\right)$, where $p\in Z^{+}$, along with an initial quantum state $|S_{0}\rangle$, we prepare a parameterized quantum circuit that transforms $|S_{0}\rangle$ by 2*p* operators in form of $e^{-i\gamma_{j}H_{P}}$ and $e^{-i\beta_{j}H_{B}}$ with j∈[p]. The final quantum state, obtained by this circuit, can be written as:

$$|\gamma ,\beta \rangle =e^{-i\beta_{p}H_{B}}e^{-i\gamma_{p}H_{P}}\ldots e^{-i\beta_{1}H_{B}}e^{-i\gamma_{1}H_{P}}|S_{0}\rangle$$

 |γ,β〉 is the distribution of all potential solutions for the problem, depending on parameters (γ,β).

*Step 4*: We use a classic optimizer to find the optimal parameters $\left(\gamma^{*},\beta^{*}\right)$ such that the expectation $\langle \gamma^{*},\beta^{*}|H_{P}|\gamma^{*},\beta^{*}\rangle$ is minimum.

### 2.2 Problem definition

Given a set of regulatory interactions among genes as D (e.g. D={”Activation”,”Repression”}), we model a regulatory network as a connected graph G=(V,E,φ), where V is the set of nodes, E is the set of edges, and φ:E→D is a mapping from an edge to its corresponding regulatory relationship. We define a motif pattern as a connected graph $M=(V^{\prime },E^{\prime },\varphi^{\prime })$, where $V^{\prime }$, $E^{\prime }$, and $\varphi^{\prime }:E^{\prime }\to D$ represent the set of motif nodes, edges, and the mapping from edges to regulatory relationships, respectively. Given two graphs $G_{1}=(V_{1},E_{1},\varphi_{1})$ and $G_{2}=(V_{2},E_{2},\varphi_{2})$, we say that $G_{1}$ and $G_{2}$ are isomorphic if there exists a bijection (one-to-one and onto mapping) $g:V_{1}\to V_{2}$ such that for every pair of nodes $u,v\in V_{1}$, we have the edge $(u,v)\in E_{1}$ iff the edge $(g(u),g(v))\in E_{2}$ and the regulatory relationships between two edges are same (i.e. $\varphi_{1}((u,v))=\varphi_{2}((g(u),g(v))$). We say that a subset of edges ϒ⊆E, is an embedding of the motif pattern M in G, if the induced subgraph G[ϒ] is isomorphic to M, denoted by G[ϒ]≡M. We consider two embeddings ${ϒ}_{1}$ and ${ϒ}_{2}\subseteq E$ to be disjoint if the induced subgraphs $G[{ϒ}_{1}]$ and $G[{ϒ}_{2}]$ do not share any nodes.

For a given set of embeddings W={ϒ|ϒ⊆E,G[ϒ]≡M}, we introduce the function ϕ, defined as $\phi (W)=\cup_{ϒ\in W}ϒ$. We refer to ϕ(W) as an *edge decomposition* of W. Additionally, we characterize a set of embeddings W as disjoint if $\forall {ϒ}_{i}$ and ${ϒ}_{j}\in W$, $|{ϒ}_{i}\cap {ϒ}_{j}|=\emptyset$. We now formally define of the MI problem as follows:

Definition 1 **(MI problem)** Consider a network G=(V,E,φ) and a motif pattern $M=(V^{\prime },E^{\prime },\varphi^{\prime })$. The MI problem aims to find the largest set W of disjoint embeddings of M in G.

In Definition 1, there is no specific linkage between the given elements (the network G and motif pattern M) and the task at hand (identifying the largest set of disjoint embeddings). Therefore, we examine the “disjoint” characteristic in term of given inputs through Lemmas 1 and 2. We provide proofs of all lemmas and theorems in the Supplementary Material S1.

Lemma 1 *Consider a network* G=(V,E,φ)  *and a motif pattern* $M=(V^{\prime },E^{\prime },\varphi^{\prime })$*. Given two sets of disjoint embeddings* $W_{1}$  *and* $W_{2}$  *of* M  *into* G  *such that* $W_{1}\neq W_{2}$*, then* $\phi (W_{1})\neq \phi (W_{2})$.

According to Lemma 1, we deduce that, given a set of disjoint embeddings W, the edge decomposition ϕ(W) is unique. Conversely, if we are given a set of edges E and are aware that E constitutes an edge decomposition of a disjoint embedding set W, we are able to reconstruct W. In Lemma 2, we establish properties that characterize a set of edges E as an edge decomposition.

Lemma 2 *Consider a network* G=(V,E,φ)  *and a motif pattern* $M=(V^{\prime },E^{\prime },\varphi^{\prime })$*. Given an arbitrary edge set* E={e|e∈E}*, we show that* E  *is a unique edge decomposition of a disjoint embedding set* W  *of* M  *in* G*, denoted as* E≡ϕ(W)*, if it has properties as follows:*

**Property 1:** For every e∈E, there exist a set of $|E^{\prime }|-1$ distinct edges $S_{e}=\{{\bar{e}}_{1},\ldots ,{\bar{e}}_{|E^{\prime }|-1}\in E\}$ such that $G[\{e\}\cup S_{e}]\equiv M$.

**Property 2:** For every $e_{1},e_{2}\in E$ such that $e_{1}$ and $e_{2}$ share a same node, then $e_{1}\in S_{e_{2}}$ and $e_{2}\in S_{e_{1}}$.

From these two lemmas, we rewrite the MI problem’s definition, which is equivalent to that in Definition 1 but is better aligned to the quantum solution we will develop in the rest of this section:

Definition 2 **(MI-alternative)** Consider a network G=(V,E,φ) and a motif pattern $M=(V^{\prime },E^{\prime },\varphi^{\prime })$. The MI problem aims to find the largest set of edges $E=\{e_{i}|e_{i}\in E\}$ such that E satisfies the two properties in Lemma 2.

### 2.3 Integer representation for the MI problem

We model two regulatory relationships consisting of activation and repression, which we label as 0 and 1, respectively. Thus, the set of regulatory relationships D is {0,1}. Given network G=(V,E,φ) and motif $M=(V^{\prime },E^{\prime },\varphi^{\prime })$, we use the term (i,j) with i,j∈V and $\left(i^{\prime },j^{\prime }\right)$ with $i^{\prime },j^{\prime }\in V^{\prime }$ to denote an edge in G and M, respectively. Additionally, given two nodes i,j∈V, φ(i,j)=0 if (i,j)∈E and the relationship is activation, while φ(i,j)=1 if (i,j)∈E and the relationship is repression. In case (i,j)∉E, given a large constant Ω, φ(i,j)=Ω. The same rules are applied to $\varphi^{\prime }$. Given two nodes i,j∈V and two nodes $i^{\prime },j^{\prime }\in V^{\prime }$, we define $c_{iji^{\prime }j^{\prime }}=1-\left|\varphi (i,j)-\varphi^{\prime }(i^{\prime },j^{\prime })\right|$. We notice that $c_{iji^{\prime }j^{\prime }}=1$ if (i,j) and $\left(i^{\prime },j^{\prime }\right)$ have the same regulatory relationship, or if both (i,j)∉E and $(i^{\prime },j^{\prime })\notin E^{\prime }$.

We model edges selected to be in the solution (i.e. resulting motifs) with a set of binary variables $X=\{x_{ij}|(i,j)\in E\}$. More specifically, we denote edge (i,j) with $x_{ij}$ as:

$$x_{ij}=\{\begin{array}{cc} 0 & \text{if}\;(i,j)\;\text{is}\;\text{not}\;\text{selected}.\\ 1 & \text{if}\;(i,j)\;\text{is}\;\text{selected}. \end{array}$$

Let us denote $n=|V^{\prime }|$. Without loss of generality, we assume that nodes in the motif M are labeled from 1 to n and there always exists an edge between nodes 1 and 2. We realize that an embedding of M in G is equivalent to a n-permutation of the set V. Specifically, we present a n-permutation as $P=[\pi_{1},\pi_{2},\ldots ,\pi_{n}]$ where $\pi_{i}$ corresponds to the node i∈M. In addition, given two nodes i,j∈V and a set S⊆V, we define the set of possible n-permutations which have $\pi_{1}=i$, $\pi_{2}=j$ and for k∈[3,n], $\pi_{k}\in S\setminus \{i,j\}$ as $P_{ij}^{S}$. We notice that given (i,j)∈E, each n-permutation $P=[\pi_{1},\ldots ,\pi_{n}]\in P_{ij}^{S}$ corresponds to a distinct edge set $E_{P}=\{(\pi_{i^{\prime }},\pi_{j^{\prime }})|(i^{\prime },j^{\prime })\in E^{\prime }\}$. Thus, given an n-permutation P, if all edges in the set $E_{P}$ are selected (i.e. $x_{\pi_{i^{\prime }}\pi_{j^{\prime }}}=1,\;\forall (i^{\prime },j^{\prime })\in E^{\prime }$) and $G[E_{P}]\equiv M$, we have

$$\prod_{(i^{\prime },j^{\prime })\in E^{\prime }}x_{\pi_{i^{\prime }}\pi_{j^{\prime }}}c_{\pi_{i^{\prime }}\pi_{j^{\prime }}i^{\prime }j^{\prime }}=1$$

As a result, given the edge (i,j)∈E, the number of embeddings in G formed by an n-permutation in $P_{ij}^{S}$ is

$$h_{ij}^{S}=\sum_{[\pi_{1},\ldots ,\pi_{n}]\in P_{ij}^{S}}\prod_{(i^{\prime },j^{\prime })\in E^{\prime }}x_{\pi_{i^{\prime }}\pi_{j^{\prime }}}c_{\pi_{i^{\prime }}\pi_{j^{\prime }}i^{\prime }j^{\prime }}$$

Furthermore, given an edge (i,j)∈E, we define the set nodes excluding *i*, *j* as $V_{ij}\equiv V\setminus \{i,j\}$ and the set of adjacent edges of (i,j) as $A_{ij}$. In accordance with Definition 2, based on above definitions, we formulate the MI problem as a constrained integer model as:

*Maximize:*
 

$$\sum_{(i,j)\in E}x_{ij}$$

*Subject to:*
 

(1) $$\begin{array}{c} x_{ij}-h_{ij}^{V}=0\;\forall (i,j)\in E \end{array}$$

 

(2) $$\begin{array}{c} x_{ij}x_{kt}(h_{ij}^{V_{kt}}+h_{kt}^{V_{ij}})=0 \end{array}$$

 

(3) $$\begin{array}{c} \forall (i,j)\in E,(k,t)\in A_{ij}\\ x_{ij}=0\;\forall (i,j)\notin E \end{array}$$

The formulation above maximizes the number of selected edges such that these edges can form a set of disjoint embeddings. These three constraints follow the properties of disjoint embedding sets, as established in Lemma 2. Constraint (1) ensures that for each selected edge (i,j)∈E, there exists exactly one distinct selected edge set that is isomorphic to the motif M with i and j as two first nodes in M. This constraint corresponds to the first property in Lemma 2. Constraint (2) ensures that for every pair of selected edges (i,j),(k,t)∈E which are adjacent edges, there is no motif constructed by n-permutations of $P_{ij}^{V_{kt}}$ and $P_{kt}^{V_{ij}}$. This constraint corresponds to the second property in Lemma 2. Finally, Constraint (3) assigns $x_{ij}=0$ if the given network G does not contain the edge (i,j).

Lemma 3 *An assignment of values to the variables in* X  *which maximizes the number of edges and satisfies three constraints (1), (2), and (3) in our integer model above yields the optimal solution to the MI problem.*

Following from this lemma, to design a quantum solution for the MI problem, given a set of variables X, we represent the integer model of the MI problem as an unconstrained objective function f:X→R. The function f includes a cost function $f_{c}$ which evaluates the quality of the input X (i.e. the number of edges) and three penalty functions $f_{p_{1}}$, $f_{p_{2}}$, and $f_{p_{3}}$ which validate the input X in term of Constraints (1), (2), and (3), respectively. In details, we have:

(4) $$f(X)=-f_{c}(X)+f_{p_{1}}(X)+f_{p_{2}}(X)+f_{p_{3}}(X)$$

The target function $f_{c}(X)=\sum_{(i,j)\in E}x_{ij}$ is equivalent to the target of the integer model. $f_{c}(X)$ returns the number of selected edges in X. The penalty function $f_{p_{1}}$ ensures that the assignment X satisfies Constraint (1). $f_{p_{1}}(X)$ returns 0 if X satisfies Constraint (1), and returns a large number otherwise. Given a large constant $\Omega_{1}$, we compute the first penalty function as:

(5) $$f_{p_{1}}(X)=\Omega_{1}\sum_{(i,j)\in E}{\left(x_{ij}-h_{ij}^{V}\right)}^{2}$$

Similarly, given a large constant $\Omega_{2}$, we compute the second penalty function as:

(6) $$f_{p_{2}}(X)=\Omega_{2}\sum_{(i,j)\in E,(k,t)\in A_{ij}}x_{ij}x_{kt}{\left(h_{ij}^{V_{kt}}+h_{kt}^{V_{ij}}\right)}^{2}$$

Finally, given a large constant $\Omega_{3}$, we compute the third penalty function as:

(7) $$f_{p_{3}}(X)=\Omega_{3}\sum_{(i,j)\notin E}x_{ij}$$

 

Theorem 1 *The assignment of* X*, which minimizes the function* f*, optimally solves the MI problem.*

### 2.4 A quantum circuit design for the MI problem

Here, we provide a detailed description of the quantum circuit which QAOA employs to solve the MI problem. Given the cardinality of the set X as r=|X|, the circuit is designed to operate on a r−qubit system. Specifically, each assignment of X corresponds to a basis state in the r−qubit system. As the mixing Hamiltonian is fixed, we need to construct the initial state $|S_{0}\rangle$, and the problem Hamiltonian $H_{P}$ for the circuit.

First, we define the initial state $|S_{0}\rangle$ used in QAOA as a superposition of all possible basis states with equal amplitudes. $|S_{0}\rangle$ can be expressed as:

$$|S_{0}\rangle =(|0\rangle +|1\rangle {)}^{⊗r}$$

We define the problem Hamiltonian $H_{P}$ which encodes objective function f such that $H_{P}|X\rangle =f(X)|X\rangle$. Given a variable x∈X, we define $Z^{\left(x\right)}$ as the Pauli-Z gate that acts on the qubit corresponding to x. Given the identity operator I, we can construct $H_{P}$ by substituting each variable x∈X in the objective function f as $\frac{1}{2}(I-Z^{\left(x\right)})$ (Hadfield 2021). By measuring this circuit, we can obtain a quantum state that represents the distribution of potential solutions for the MI problem.

## 3 Discussion

**Figure 1.**
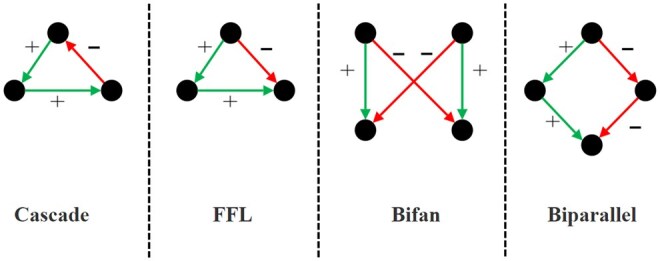
Four possible motif patterns with corresponding regulatory relations used in the synthetic dataset. The green color (or +) represents activation, while the red one (or −) represents repression. The distribution of the activation and repression to the motif edges show one possible configuration. Other configurations with different number and distribution of activation and repressions are also possible.

In this section, we assess the performance of QOMIC using synthetic and real datasets. We focus on four motif topologies, namely cascade, feed forward loop (FFL), bifan, and biparallel, which occur frequently in biological networks (Milo *et al.* 2002). For each motif, we consider common regulatory relationships reported in the literature (Ingram *et al.* 2006, Kim *et al.* 2008, Papatsenko 2009). Figure 1 depicts the motifs and their regulatory relationships.

*Datasets*: We use synthetic and real datasets in our experiments.

*Synthetic datasets:* In order to examine the performance of QOMIC under networks with diverse topological properties, we conduct benchmarking experiments using synthetic datasets. The properties that govern synthetic datasets are as follows. First, we define the number of nodes and the average degree of nodes as n and d, respectively. These two parameters control the size and density of the network. We define the ratio of activation interactions in a network as r. Specifically, the ratio of activation interactions is calculated as the number of activation interactions divided by the total number of edges. This parameter influences the distribution of activation and repression interactions. We construct a synthetic network by first creating a number of predefined motifs of a given motif topology. Given a edge ratio *edr* randomly selected in range of [0.2, 0.6], the number of predefined motifs is calculated as $\left\lceil \left(d\times \mathrm{edr}\times n)/(2\times |E^{\prime }|\right)\right\rceil$. For example, we consider to generate a network with n=600 nodes, and average node degree d=4. Assume that we want this network includes FFL motifs and 10% of the edges in this network belong to FFL motifs (that is edr= 0.1). 10% of the network’s edges are $\frac{n\times d\times \mathrm{edr}}{2}=1200\times 0.1=$ 120. Since each instance of the FFL motif has three edges ($|E^{\prime }|=3$), the total number of motifs we embed is $\frac{n\times d\times \mathrm{edr}}{2\times |E^{\prime }|}=120/3=40$. We note that predefined motifs can be overlapped, so the number of predefined motifs cannot be considered as the solution for our MI problem.

We then randomly insert edges and their regulatory interactions until the network size, density, and ratio of activation constraints are satisfied. We generate different networks by varying these parameters as: n∈{200,400,600,800,1000}, d∈{2,4,6,8,10}, r∈{0.2,0.5,0.8} for each of the four motif topologies shown in Fig. 1. Note that, for each motif topology, there are alternative configurations by varying the distribution of activation/repression relationships to the edges. We use the configuration in Fig. 1 to create this dataset as the rest of the algorithm does not depend on this configuration. For each combination of these parameters, we generate five synthetic networks. In total, we have 5×5×3×4×5=1500 synthetic networks.

*Real datasets:* In order to evaluate the performance of QOMIC on real datasets, we use the TRRUST dataset (Han *et al.* 2017). TRRUST is a manually curated database of human and mouse transcriptional regulatory networks. We use the human networks from this dataset. The total number of human transcriptional regulatory interactions in this dataset is 8427, with 2428 activation, 1581 repression, and 4418 unknown interactions. We focus on neurodegenerative diseases, specifically Alzheimer’s, Parkinson’s, Huntington’s, Amyotrophic Lateral Sclerosis (ALS), and Motor Neurone Disease (MND). Through DisGeNet, we find the Gene-Disease Associations to find which genes are related to the listed diseases, with correlation being given through a score from 0 to 1. In this work, we set the correlation threshold to select genes as 0. Details of genes correlated to five diseases are provided in Supplementary Material S2. We then tailor our TRRUST dataset to include only genes that are associated with the specific diseases being investigated. The details of real datasets are summarized in Supplementary Section SM 2 (Supplementary Material S1).

*The baseline methods*: We compare QOMIC against three baseline methods including Ren *et al.* (2019), *FASE* (Paredes and Ribeiro 2015), and *Mfinder* (Milo *et al.* 2002). The Ren *et al.* method systematically identifies motif embeddings within the network by computing the loss value, which indicates of the number of embeddings that cannot be concurrently selected with the current one, and then iteratively selecting embeddings with minimal loss until independent embeddings are exhausted. FASE is an approximation method which utilizes the G-Trie structure to efficiently store and count the number of subgraphs. Mfinder uses a semidynamic programming algorithm for enumerating motif embeddings.

*Implementation*: In the integer model for the MI problem, the number of required qubits corresponds to the number of edges in the network G. Due to the limitation on the number of qubits in current quantum machines, we employ a partitioning technique to address the large networks. In details, we divide the initial network G into a collection of sub-networks, and then apply QOMIC on each sub-networks. Finally, we aggregate resulting motifs on sub-networks to derive the total motifs presented in the initial network G. Our partitioning technique ensures that motifs in the final solution are pairwise disjoint. In addition, we implement and test QOMIC using IBM quantum simulators (Aleksandrowicz *et al.* 2019). Our implementation can be found in https://github.com/ngominhhoang/Quantum-Motif-Identification.git.

### 3.1 Evaluation on synthetic datasets

We benchmark the performance of QOMIC and three baseline methods on different criteria of 1500 synthetic datasets including network size, network density, and the distribution of regulatory relationships. The network size, density, and ration of activation of those networks are described in the previous part. For each experiment, we compare four methods in terms of the number of resulting motifs. The time limit set for each test instance is 3600 s (1 h). For most of test instances related to the bifan and biparallel motifs, Mfinder exceeds the time limit without returning any feasible solution, so we do not report the results of Mfinder for the bifan and biparallel. We report detailed running time results in Supplementary Section SM 4 (Supplementary Material S1).

**Figure 2.**
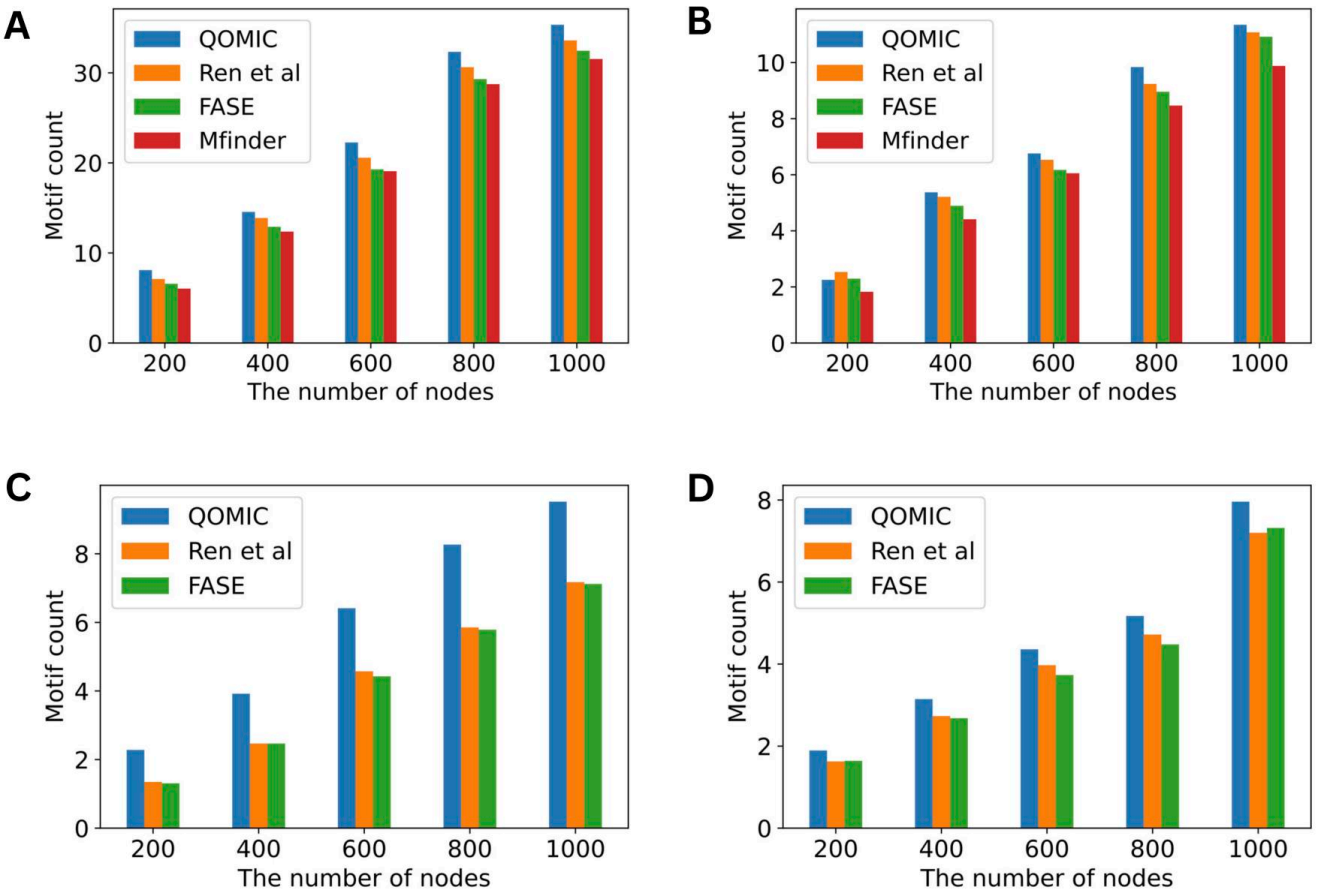
Analysis of the QOMIC and three baseline methods in term of the number of motif embeddings found by varying the number of nodes in the synthetic networks. The analysis is illustrated in four motif types including (A) Cascase, (B) FFL, (C) Bifan, and (D) Biparallel.

*The impact of network size*: In this experiment, we compare the performance of four methods under different network sizes ranging from 200 to 1000 nodes. Figure 2 shows the average number of motifs resulting from QOMIC and baseline methods for each network size. We observe that QOMIC consistently outperforms all baseline methods in identifying all four motif types (19/20 comparisons). Specifically, the number of cascade, FFL, bifan, and biparallel motifs detected by QOMIC exceed those found by the second best method, Ren *et al.*, by 6.2%, 3.4%, 41.9%, and 6.9%, respectively. Additionally, the disparity in solution quality between QOMIC and three other methods becomes more significant in the case of the bifan and biparallel motifs which possess more complex topologies compared to the cascade and FFL motifs. On the other hand, it is significant to note that cascade motifs are more prevalent than the other three motif types at the same network size. This occurrence can be attributed to the cyclic topology of the cascade motif, which relaxes constraints on the regulatory relationships within the motif. Finally, as the network size increases, the gap between QOMIC and three baseline methods grows in favor of our method. Thus, QOMIC is even more advantageous when dealing with complex motif topologies and large networks.

**Figure 3.**
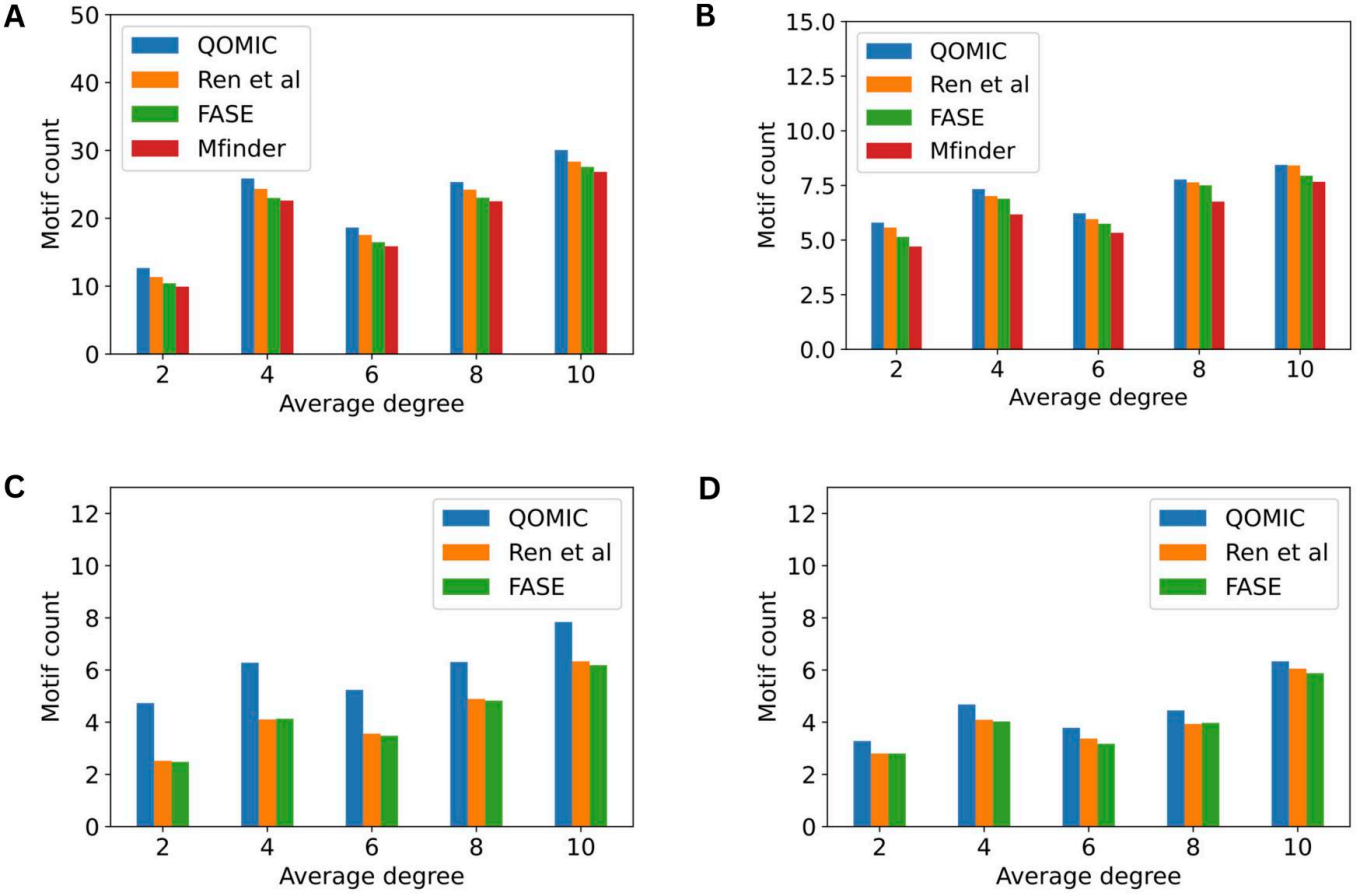
Analysis of the QOMIC and three baseline methods in term of the number of motif embeddings found by varying the density in the synthetic networks. The analysis is illustrated in four motif types including (A) Cascase, (B) FFL, (C) Bifan, and (D) Biparallel.

*The impact of network density*: Next, we compare four methods by varying the density of networks from 2 to 10. Figure 3 illustrates the average number of motifs obtained using QOMIC and baseline methods. Similar to the previous results, QOMIC provides superior solutions than the baseline methods in all cases (20/20 comparisons). However, unlike the previous findings where the motif count increased with the number of nodes, it is noteworthy that networks with higher average degree may yield fewer motifs. Specifically, in four cases of motif types, the number of motifs found in networks with a density of 6 is lower than in networks with a density of 4. This is due to the fact that additional edges might lead to overlapping motifs which violate the constraint of the MI problem. Consistent with our previous results, we observe more gain in motif count using our method for complex motif topologies, such as bifan.

**Figure 4.**
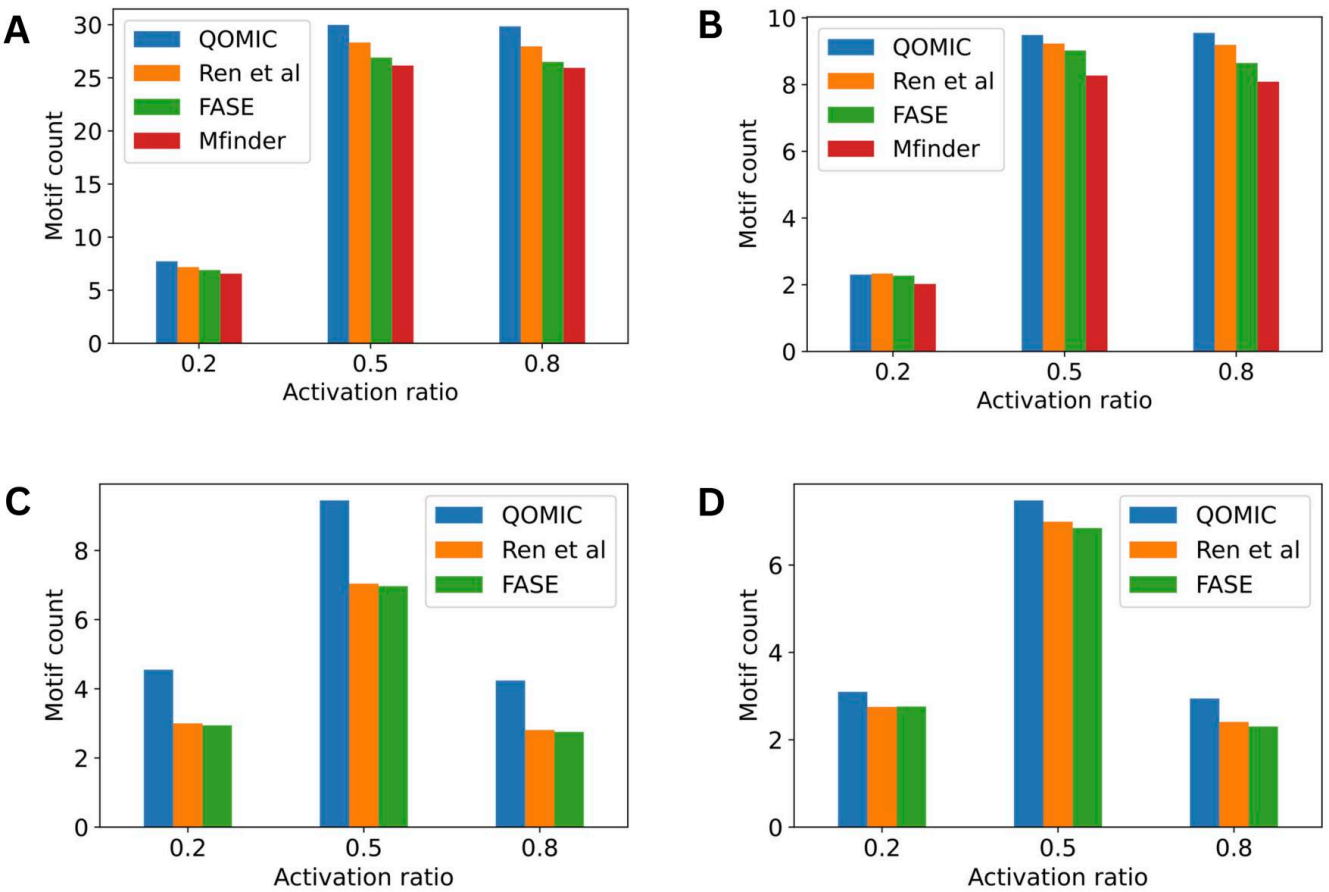
Analysis of the QOMIC and three baseline methods in term of the number of motif embeddings found by varying the activation ratio in the synthetic networks. The analysis is illustrated in four motif types including (A) Cascase, (B) FFL, (C) Bifan, and (D) Biparallel.

*The impact of regulatory ratio*: Then, we consider the distribution of regulatory relationships in networks. In detail, we examine the ratio of activation relationships as 0.2, 0.5, and 0.8. As this ratio gets close to 0.5, the interaction types get more heterogeneous. Figure 4 presents the average number of motifs obtained using QOMIC and baseline methods. We observe that QOMIC identifies more motifs than other methods in 11/12 cases. Additionally, for cascade and FFL motifs, networks with activation probabilities of 0.5 and 0.8 exhibit a notably higher motif count compared to networks with a 0.2 ratio. On the other hand, for the bifan and biparallel motifs, the motif count of networks with an activation probability of 0.8 surpasses the motif count of networks with two other ratios. These observations suggest that networks which have similar activation ratio with the ratio of the target motif are more likely to contain valid embeddings.

### 3.2 Evaluation on real datasets

Here, we discuss the efficiency of QOMIC on five human regulatory networks. These network includes genes related to five neurodegenerative diseases: Alzheimer’s, Parkinson’s, Huntington’s, ALS, and MND. Motif patterns we aim to identify include cascade, FFL, bifan, and biparallel. In this experiments, information about regulatory interactions is incomplete, as some regulatory interactions have not yet been discovered in the literature. Thus, our method shifts the goal to identifying embeddings without considering the similarity of regulatory interactions between the found embeddings and the given motif by setting $c_{iji^{\prime }j^{\prime }}=1\forall i,j\in V,i^{\prime },j^{\prime }\in V^{\prime }$ in our formulation. We report a detailed analysis of the statistical significance of our results in Supplementary Section SM 3.

*Motif distribution*: Table 1 presents the number of motifs identified for each pattern and disease, along with the number of edges with activation and repression relations in these motifs. For instance, in the Alzheimer’s-related network, QOMIC identified seven cascade motifs. Among the edges of these motifs, there are three activation relations and four repression relations. The activation/repression status of the remaining 14 interactions is unknown. Further details on this example can be found in our Supplementary Material S1, Section SM 3. In general, among four motif patterns, the cascade pattern contributes the fewest number of motifs, accounting for only 5.4% of the total, while the bifan pattern contributes the most number of motifs, with 44.3% in total. This observation suggests that in regulatory networks associated with five diseases, the bifan topology is the most prevalent, whereas the triangle loop topology (cascade) is relatively rare. In addition, we observe that among five diseases, networks associated with Alzheimer’s and Parkinson’s contribute nearly 60% of the total number of motifs, while the network of MND only contributes roughly 2%. This phenomenon suggests that genes related to Alzheimer’s and Parkinson’s exhibit strong regulatory relationships with each other by four popular motif patterns. On the other hand, among the motifs discovered, the total count of activation relations is about 1.3 times greater than the total count of repression relations. This ratio is consistent with the ratios observed in synthetic motif patterns. Furthermore, the sum of activation and repression counts is moderately smaller than the total number of edges within the motifs found in nearly all cases. This is because of a large number of relationships between genes being categorized as unknown. On average, each motif embedding found includes ∼50% of unknown edges.

**Table 1.** The statistics on the motif count (#) per patterns found by QOMIC in five disease-related regulatory networks.^a^

	Cascade			FFL			Bifan			Biparallel		
	#	+	−	#	+	−	#	+	−	#	+	−
Alzheimer’s	7	3	4	52	43	37	72	85	57	27	27	25
Parkinson’s	5	6	2	48	36	27	55	62	36	22	19	18
Huntington’s	7	4	5	32	24	26	41	51	30	12	13	13
ALS	6	2	1	32	17	24	40	41	29	12	15	8
MND	1	1	1	3	1	3	5	6	6	1	0	0

a The columns shown with + and − indicate the number of activation and repression interactions in resulting motifs. The activation/supression status of the remaining edges in the resulting motifs are unknown.

**Figure 5.**
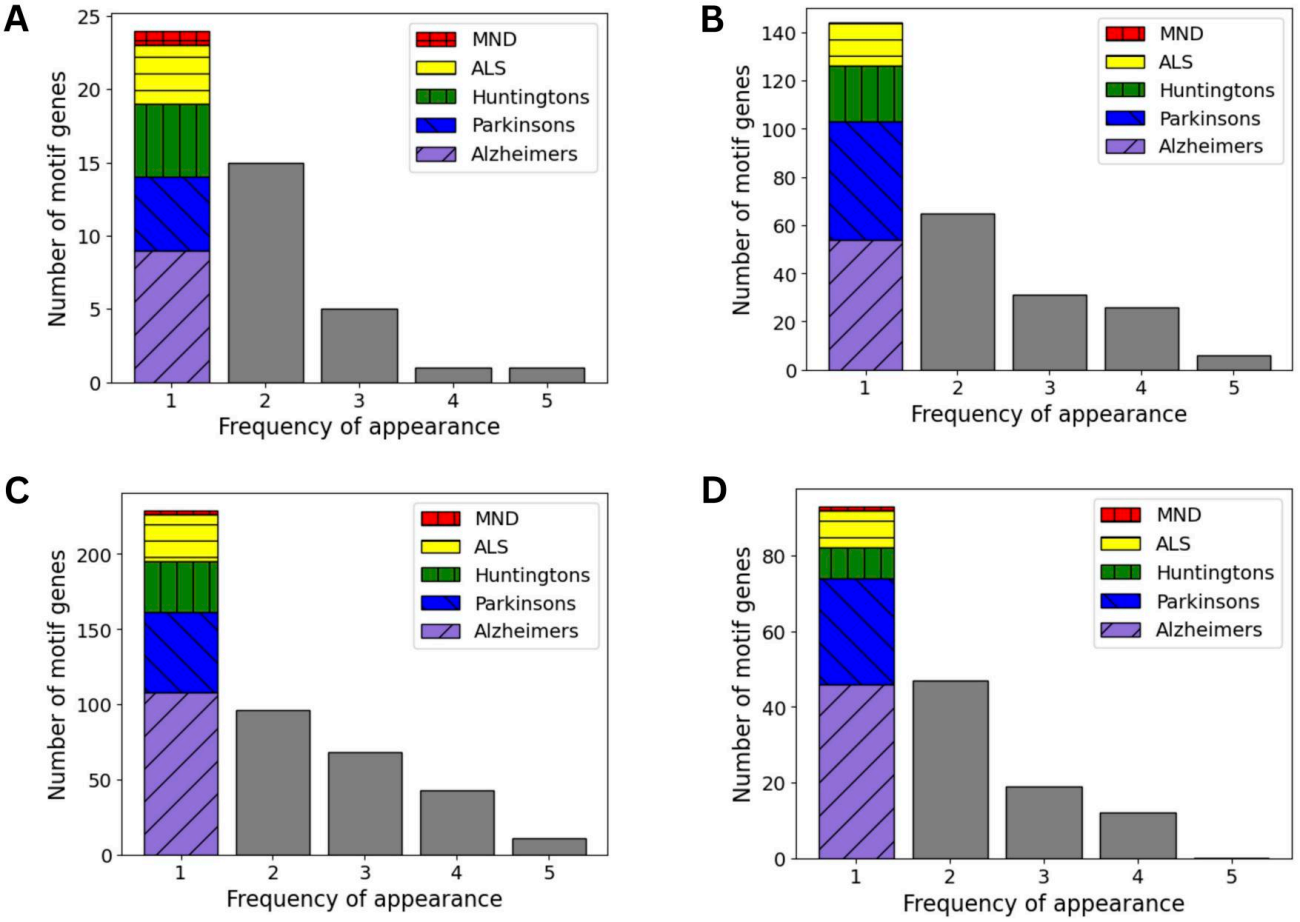
The statistics on the number of motif genes with different appearance frequency in five diseases. The first column represents the number of genes exclusively linked to one of five diseases. The statistics are illustrated in four motif types including (A) Cascase, (B) FFL, (C) Bifan, and (D) Biparallel.

*Distribution of motif genes across diseases*: Here, we invest in the appearance of genes in motifs found. We denote a gene associated with a motif as *motif gene*. Figure 5 illustrates the number of motif genes appeared with different frequency in five diseases. In all four motifs, the number of motif genes is inversely proportional to the number of appearance of motif genes in five diseases. Specifically, the number of motif genes included in exact one disease is even more than the total number of motif genes included in more than one disease. This observation shows that each disease includes an own set of genes which are topologically related to each others.

When we delve deeper into the proportions of these unique genes to each disease, we find out that motif genes uniquely related to Alzheimer’s and Parkinson’s account for more than 60% in total number of unique motif genes for all motif types. However, not every disease owns a strong set of unique motif genes. Specifically, the number of motif genes exclusively linked to the MND disease is fewer than the number of motif genes related to all diseases in most cases of motif patterns. We infer that motif genes related to the MND disease may have a broader relevance, because they are also associated with various other diseases.

*Further findings*: We analyze the motifs identified by QOMIC and perform gene enrichment analysis for each of the five neurodegenerative diseases in Supplementary Material S1 (Supplementary Section SM 2). We observe that each motif can identify a unique set of molecular functions with very low false discovery rates. Our statistical significance analysis (see Supplementary Material S1, Section SM 3) demonstrates that FFL motif yields very high statistical significance for Alzheimer’s, Parkinson’s, Huntingtons’s, and ALS disorders; Bifan yields high statistical significance for all neurodegenrative diseases. We defer further discussion and detailed results to Supplementary Material S1.

### 3.3 Concluding remarks

Network MI problem is a significant problem in the field of biology, especially when we incorporate the $F_{3}$ measure for the target network and introduce regulatory constraints to motif patterns. However, the limited computational capacity of classical computers hinders the scalability of traditional methods for this problem. In this work, using quantum computing scheme, we propose a novel quantum solution, named QOMIC, for the MI problem. We implement and test the performance of QOMIC on the IBM’s quantum gate-based machine. Although quantum computing is in the early states of development, the experimental results on both synthetic and real datasets show that QOMIC can efficiently identify motifs within reasonable running times. In terms of motif count, QOMIC even outperforms the baseline methods in almost all cases. This suggests that quantum computing is a promising approach in solving complex biological problems in the future.

## Supplementary Material

vbae208_Supplementary_Data

## Data Availability

The datasets were derived from the following public domain resources: https://www.grnpedia.org/trrust/ and in the Supplementary Materials.
