# Supplementary material for: QOMIC: quantum optimization for motif identification
Source: Bioinform Adv. 2024 Dec 24;5(1):vbae208. doi: 10.1093/bioadv/vbae208 (PMC11725347; doi:10.1093/bioadv/vbae208)
Supplement: vbae208_Supplementary_Data [file vbae208_supplementary_data.zip › Supplementary_Material_1.pdf]

Supplementary Material

SM 1: Correctness of the QOMIC algorithm

Lemma 1 Consider a network $G = (V, E)$ and a motif pattern $M = (V', E')$. Given two sets of disjoint embeddings $\mathcal{W}_1$ and $\mathcal{W}_2$ such that $\mathcal{W}_1 \neq \mathcal{W}_2$, then $\phi(\mathcal{W}_1) \neq \phi(\mathcal{W}_2)$.

Proof We prove this lemma by contradiction. We assume that there exists two different sets of disjoint embeddings $\mathcal{W}_1$ and $\mathcal{W}_2$ such that $\phi(\mathcal{W}_1) = \phi(\mathcal{W}_2)$. Without loss of generality, we denote an embedding $\Upsilon = \{e_1, \ldots, e_{|V'|}\}$ such that $\Upsilon \in \mathcal{W}_1$ and $\Upsilon \notin \mathcal{W}_2$. Recall that we have $\phi(\mathcal{W}_1) = \phi(\mathcal{W}_2)$, so it follows that $e_1, \ldots, e_{|V'|}$ must belong to at least two different embeddings of $\mathcal{W}_2$ **(1)**.

Because we assume that the motif M is connected and have at least 3 nodes, the embedding Υ is itself a connected component with at least 3 nodes **(2)**.

From **(1)** and **(2)**, we can infer that there exists two edges in Υ that share a same node and belong to two different embeddings in $\mathcal{W}_2$. This violates the assumption of $\mathcal{W}_2$ which is supposed to contain all disjoint embeddings. Thus, given two sets of disjoint embeddings $\mathcal{W}_1$ and $\mathcal{W}_2$ such that $\mathcal{W}_1 \neq \mathcal{W}_2$, we can prove that $\phi(\mathcal{W}_1) \neq \phi(\mathcal{W}_2)$. □

Lemma 2 Consider a network $G = (V, E)$ and a motif pattern $M = (V', E')$. Given an arbitrary edge set $\mathcal{E} = \{e | e \in E\}$, we show that $\mathcal{E}$ is a unique edge decomposition of a disjoint embedding set $\mathcal{W}$ of M into G, denoted as $\mathcal{E} \equiv \phi(\mathcal{W})$, if it has properties as follows:

- **Property 1:** For every $e \in \mathcal{E}$, there exist a set of $|E'| - 1$ distinct edges $S_e = \{e_1, \ldots, e_{|E'|-1} \in \mathcal{E}\}$ such that $G[\{e\} \cup S_e] \equiv M$.
- **Property 2:** For every $e_1, e_2 \in \mathcal{E}$ such that e_1 and e_2 share a same node, then $e_1 \in S_{e_2}$ and $e_2 \in S_{e_1}$.

Proof $\mathcal{E}$ is a unique edge decomposition of a disjoint embedding set $\mathcal{W}$ of M into G if $\mathcal{E}$ satisfies two following conditions:

- **Condition 1:** There exists a unique way to completely assign every edge in $\mathcal{E}$ into distinct groups $\Upsilon_1, \ldots, \Upsilon_m$ with $m = \frac{|\mathcal{E}|}{|E'|}$ such that $G[\Upsilon_i] \equiv M$ with $i = 1, \ldots, m$.
- **Condition 2:** $\Upsilon_1, \ldots, \Upsilon_m$ are pairwisely disjoint.

We will prove that an edge set $\mathcal{E}$ with Property 1 and 2 can satisfy Condition 1 and 2 above.

With Property 1, we can establish a method for assigning edges in $\mathcal{E}$ to m distinct groups, as outlined in the first condition. To begin, we select an arbitrary edge, denoted as $e^{(1)} \in \mathcal{E}$. Then, we select a edge set $S_{e^{(1)}} = \{e_1^{(1)}, \ldots, e_{|E'|-1}^{(1)}\}$ such that $G[\{e^{(1)}\} \cup S_{e^{(1)}}] \equiv M$. The existence of $S_{e^{(1)}}$ is guaranteed by Property 1. Then, we assign $\{e^{(1)}\} \cup S_{e^{(1)}}$ as the first group Υ_1.

Moving forward, we select another arbitrary edge $e^{(2)} \in \mathcal{E} \setminus \Upsilon_1$. Similarly, we choose a edge set $S_{e^{(2)}} = \{e_1^{(2)}, \ldots, e_{|E'|-1}^{(2)}\}$ such that $G[\{e^{(2)}\} \cup S_{e^{(2)}}] \equiv M$. We assign $\{e^{(2)}\} \cup S_{e^{(2)}}$ as the second group Υ_2. Because of Property 1, we can prove that Υ_1 and Υ_2 are distinct. In other words, we demonstrate that $e_1^{(2)}, \ldots, e_{|E'|-1}^{(2)} \in \mathcal{E} \setminus \Upsilon_1$. By contradiction, we assume that there exists an edge $e' \in S_{e^{(2)}}$ such that $e' \in \Upsilon_1$. As a result, for e', there exists two different $S_{e'}$ such that $G[\{e'\} \cup S_{e'}] \equiv M$ that contradicts to Property 1. Thus, Υ_1 and Υ_2 are distinct.

By following a similar approach, given $i-1$ groups, we can form the ith group $\Upsilon_i = \{e^{(i)}\} \cup S_{e^{(i)}}$. Here, e_i is chosen such that $e_i \in \mathcal{E} \setminus \cup_{j=1}^{i-1} \Upsilon_j$ while $S_{e^{(i)}} = \{e_1^{(i)}, \ldots, e_{|E'|-1}^{(i)}\}$ satisfies $G[\{e^{(i)}\} \cup S_{e^{(i)}}] \equiv M$. Group Υ_i is distinct with $i-1$ previous groups. In the end, we can construct m groups that are the embeddings of M into G and pairwisely distinct **(1a)**.

Next, we show that the set of m groups $\mathcal{W} = \{\Upsilon_1, \ldots, \Upsilon_m\}$ constructed as above are unique. By contradiction, we assume that there exists a different set of m distinct groups $\mathcal{W}' = \{\Upsilon'_1, \ldots, \Upsilon'_m\}$ such that $\cup_{i=1}^{m} \Upsilon'_i = \mathcal{E}$ and $G[\Upsilon_i] \equiv \mathcal{E}$ for $i = 1, \ldots, m$. Additionally, because $\mathcal{W} \neq \mathcal{W}'$, there exists at least one group $\bar{\Upsilon} \in \mathcal{W}'$ such that $\bar{\Upsilon} \notin \mathcal{W}$. Given $\bar{e} \in \bar{\Upsilon}$, because $\bar{e} \in \mathcal{E} = \cup_{\Upsilon \in \mathcal{W}} \Upsilon$, there exists a group $\Upsilon \in \mathcal{W}$ such that $\bar{e} \in \Upsilon$. That contradicts to Property 1. Thus, the set of m groups $\mathcal{W} = \{\Upsilon_1, \ldots, \Upsilon_m\}$ is unique **(1b)**.

From **(1a)** and **(1b)**, we prove that if the edge set $\mathcal{E}$ has Property 1, it can satisfy Condition 1 **(1)**.

On the other hand, Property 2 implies that there is no two groups that share a same node. Thus, Condition 2 holds **(2)**.

From **(1)** and **(2)**, we prove the correctness of this lemma. □

Lemma 3 An assignment of $\mathbf{X}$ which maximizes the number of edges and satisfies three Constraints (1), (2) and (3) results in the optimal solution for the MI problem.

Table SM. 1. The summary of five real networks with the number of nodes (genes), the number of edges (interactions), the number of activation interactions, the number of repression interactions, and the number of unknown interactions.

Diseases	#Nodes	#Edges	#Activation interactions	# Repression interactions	#Unknown interactions
Alzheimer's	2525	7162	2063	1405	3694
Parkinson's	2215	5592	1614	1082	2896
Huntington's	1856	4212	1218	819	2175
ALS	1746	4330	1226	902	2202
MND	608	893	238	209	446

Proof We recall that given $i, j \in V$, each n-permutation $[\pi_1 = i, \pi_2 = j, \pi_3, \ldots, \pi_k]$ corresponds to a distinct edge set $E_P = \{(\pi_{i'}, \pi_{j'})|(i', j') \in E'\}$. Thus,

$$\prod_{(i',j')\in E'} x_{\pi_{i'}\pi_{j'}} c_{\pi_{i'}\pi_{j'}i'j'} = 1$$

if all edges in the set V_P are selected and $G[E_P] \equiv M$. As a result, given the edge $(i, j) \in E$, the sum

$$h^V_{ij} = \sum_{[\pi_1,\ldots,\pi_n]\in\ \mathcal{P}^V_{ij}} \prod_{(i',j')\in E'} x_{\pi_{i'}\pi_{j'}} c_{\pi_{i'}\pi_{j'}i'j'}$$

, is equal to the number of motifs including (i, j).

Constraint (1) is satisfied if $\forall(i, j) \in E$, $x_{ij} = h^V_{ij}$. If the edge (i, j) is not selected with $x_{ij} = 0$, Constraint (1) always holds because all products in h^V_{ij} includes x_{ij}. On the other hand, if the edge (i, j) is selected with $x_{ij} = 1$, the number of motifs including (i, j) must be 1. Thus, selected edges that satisfy Constraint (1) also satisfy Property 1 in Lemma 2 **(1)**.

When it comes to Constraint (2), it is always satisfied if the edge $(i, j) \in E$ or $(k, t) \in E$ is not selected. On the other hand, if there exists two selected edges $(i, j), (k, t) \in E$ which share at least one common node, Constraint (2) is satisfied if $h^{V_{kt}}_{ij} + h^{V_{ij}}_{kt} = 0$. In other word, there exists no motif which includes the edge (i, j), but does not include the edge (k, t), and vice versa. Thus, combining with Constraint (2) in which each selected edge must associate with one motif, we can imply that (i, j) and (k, t) must belong to a same motif in order to satisfy two these constraints. Thus, selected edges that satisfy Constraint (1) and (2) also satisfy Property 2 in Lemma 2 **(2)**.

Constraint (3) is to ensure that $x_{ij} = 0 \forall(i, j) \notin E$ **(3)**.

From **(1)**, **(2)**, **(3)**, and Lemma 2, a feasible assignment $\mathbf{X}$, that satisfies three constraints, corresponds to a valid edge decomposition of a disjoint embedding set of M into G. Thus, by finding the maximum feasible $\mathbf{X}$, we can obtain the maximum number of disjoint motifs.

□

Theorem 1 *The assignment of* $\mathbf{X}$*, which minimizes the function* f*, optimally solve the* MI *problem.*

Proof From the definition of the function f, a feasible $\mathbf{X}$, which satisfies three constraints in the integer model, lead to minimum values of 0 in penalty terms including f_{p_1}, f_{p_2}, and f_{p_3}. Besides, f_c corresponds to the number of selected edges from $\mathbf{X}$ with a negative sign. Consequently, $\mathbf{X}$, which minimizes the function f, represents a maximum feasible solution for the integer model. From Lemma 3, we can conclude that $\mathbf{X}$, which minimizes the function f, is the optimal solution for the MI problem. □

SM 2: The enrichment analysis corresponding to five neurodegenerative disorders

First, we summarize the information of five disease-related networks in Table SM. 1. Then, we represent the enrichment analysis of unique motif genes associated with the Alzheimer's disease. Our enrichment analysis is conducted using the GO software (Ashburner and et al, 2000; Consortium et al., 2023; Thomas and et al, 2022).

Table SM. 2 represents the top three molecular functions with the lowest false detection rates (FDR) corresponding to sets of motif genes which are uniquely associated with the Alzheimer's disease. We observe that the FDRs of all molecular functions are small (less than 10^{-6}), so sets of unique motif genes are strongly relevant to the molecular functions found. Specifically, unique motif genes of three-node patterns, including cascade and FFL, are relevant to DNA-binding functions. On the other hand, unique motif genes of four-node patterns, such as bifan and biparallel, are associated with transcription activities.

Table SM. 3 represents the top three molecular functions with the lowest FDRs corresponding to sets of motif genes associated with Parkinson's disease. Interestingly, the FDR for the cascade motif pattern is significantly higher (less than 10^{-2}), the FDR for the FFL motif is significantly lower (less than 10^{-10}), while bifan and biparallel remain in between these extremes (less than 10^{-6} and less than 10^{-5}, respectively).

Motif	Term ID	Term description	FDR
Cascade	GO:0140297	DNA-binding transcription factor binding	6.19e-09
	GO:0061629	RNA polymerase II-specific DNA-binding transcription factor binding	3.67e-08
	GO:0000978	RNA polymerase II cis-regulatory region sequence-specific DNA binding	1.99e-06
FFL	GO:0140110	Transcription regulator activity	2.45e-10
	GO:0000978	RNA polymerase II cis-regulatory region sequence-specific DNA binding	3.60e-10
	GO:0000977	RNA polymerase II transcription regulatory region sequence-specific DNA binding	4.78e-10
Bifan	GO:0000976	Transcription cis-regulatory region binding	4.89e-11
	GO:1990837	Sequence-specific double-stranded DNA binding	4.89e-11
	GO:0140110	Transcription regulator activity	5.13e-11
Biparallel	GO:0140110	Transcription regulator activity	2.61e-11
	GO:0008134	Transcription factor binding	2.72e-11
	GO:0140297	DNA-binding transcription factor binding	3.64e-10

Table SM. 2. The enrichment analysis in term of molecular functions corresponding to motif genes from the Alzheimer's-related network

Motif	Term ID	Term description	FDR
Cascade	GO:0000977	RNA polymerase II transcription regulatory region sequence-specific DNA binding	0.008
	GO:0000981	DNA-binding transcription factor activity, RNA polymerase II-specific	0.008
	GO:0000978	RNA polymerase II cis-regulatory region sequence-specific DNA binding	0.0292
FFL	GO:0000976	Transcription cis-regulatory region binding	3.81e-12
	GO:0000978	RNA polymerase II cis-regulatory region sequence-specific DNA binding	9.63e-12
	GO:0000977	RNA polymerase II transcription regulatory region sequence-specific DNA binding	1.7e-11
Bifan	GO:0000977	RNA polymerase II transcription regulatory region sequence-specific DNA binding	4.6e-07
	GO:0000978	RNA polymerase II cis-regulatory region sequence-specific DNA binding	4.6e-07
	GO:0003690	Double-stranded DNA binding	4.6e-07
Biparallel	GO:0043565	Sequence-specific DNA binding	5.42e-06
	GO:0000978	RNA polymerase II cis-regulatory region sequence-specific DNA binding	6.28e-06
	GO:1990837	Sequence-specific double-stranded DNA binding	7.03e-06

Table SM. 3. The enrichment analysis in term of molecular functions corresponding to motif genes from the Parkinson's-related network

Motif	Term ID	Term description	FDR
Cascade	GO:1990841	Promoter-specific chromatin binding	3e-06
	GO:0019899	Enzyme binding	0.0131
	GO:0019901	Protein kinase binding	0.0131
FFL	GO:0005515	Protein binding	0.005
Bifan	GO:0042802	Identical protein binding	0.0275
	GO:0140297	DNA-binding transcription factor binding	0.0419
	GO:0005102	Signaling receptor binding	0.0459
Biparallel	GO:0000978	RNA polymerase II cis-regulatory region sequence-specific DNA binding	0.0024
	GO:0000981	DNA-binding transcription factor activity, RNA polymerase II-specific	0.0024
	GO:0140110	Transcription regulator activity	0.0024

Table SM. 4. The enrichment analysis in term of molecular functions corresponding to motif genes from the Huntington's-related network

For Huntington's disease (Table SM. 4), only one molecular function corresponding to FFL was found, while the other motif patterns had at least three molecular functions. These FDRs are noticeably higher than Alzheimer's and Parkinson's, with all but one being less than 10^{-2}. Bifan in particular struggled, with all FDRs less than 10^{-1}.

In case of the ALS disease (Table SM. 5), only the FFL motif pattern has at least 3 molecular functions corresponding to ALS. Bifan only had one and the other motif patterns had none. The FDRs for the top three molecular functions of FFL are less than 10^{-2}, and the FDR for the sole bifan molecular function is less than 10^{-1}.

For the MND disease (Table SM. 6), only the biparallel motif pattern has corresponding molecular functions in MND. However, its top three FDRs are incredibly low, with all being less than 10^{-5}.

SM 3: Evaluation of the statistical significance of our results.

Here, we examine whether the number of motif embeddings found in real datasets is significant, using the concept of z-score. In order to set up the experiment, given a real target network G with the number of motif embeddings found

Motif	Term ID	Term description	FDR
FFL	GO:0000987	Cis-regulatory region sequence-specific DNA binding	0.002
	GO:0003682	Chromatin binding	0.002
	GO:0008134	Transcription factor binding	0.002
Bifan	GO:0005178	Integrin binding	0.0263

Table SM. 5. The enrichment analysis in term of molecular functions corresponding to motif genes from the ALS-related network

Motif	Term ID	Term description	FDR
Biparallel	GO:0046332	SMAD binding	3.46e-11
	GO:0070411	I-SMAD binding	5.33e-10
	GO:0005160	Transforming growth factor beta receptor binding	1.75e-6

Table SM. 6. The enrichment analysis in term of molecular functions corresponding to motif genes from the MND-related network

	AD	PD	HD	ALS	MND
Cascade	-0.26	-0.08	1.62	0.50	-0.05
FFL	6.93	8.81	5.90	5.02	-0.01
Bifan	14.58	14.04	14.63	11.15	4.01
Biparallel	-0.44	0.92	-0.21	-1.11	-0.88

Table SM. 7. The z-scores that represent statistical significance of the motif count of 4 motif patterns in 5 real regulatory networks associated with neurodegenerative diseases (AD = Alzheimer's, PD = Parkinson's, HD = Huntington Disease).

m_G, we generate N same-size random graphs $G_1, \ldots, G_N$ by randomly shuffling the edge set of G. Then, we calculate the mean, denoted as μ, and the standard deviation, denoted as σ, of the number of motif embeddings of N random graphs. Finally, we calculate the z-score as $Z = \frac{m_G - \mu}{\sigma}$. A motif pattern in a real network G is over-represented or under-represented if the z-score is greater than 2 or less than -2 respectively.

Table SM. 7 shows the z-scores of four motifs patterns in real networks corresponding to five diseases. We observe that the FFL and bifan patterns are over-represented in 4 and 5 over five disease-related networks respectively with high z-scores. Specifically, the z-scores of the FFL and bifan in over-representation cases are from 2 to 7.29 times higher than the threshold for being considered over-represented. It indicates that the presence of the FFL and bifan patterns in disease-related networks is statistically significant. In contrast, with the cascade and biparallel patterns, we find out that in all cases, their z-scores do not exceed 2 or fall below -2. It means that the occurrence of these two motif patterns are not significant in disease-related networks. From these results, we can infer that motif patterns, in which nodes are associated to either activation or repression, appear more frequently than expected in human regulatory networks related to diseases.

We also provide an illustrative example of cascade embeddings found by QOMIC in Alzheimer's regulatory network reported in Table 1. Detailed information on genes and their corresponding regulatory relationships is shown in Figure SM. 1. It is important to note that regulatory interactions reported in some practical regulatory networks are incomplete. For example, in Alzheimer's regulatory network, only one embedding with complete regulatory information (i.e., TP53 - KLF2 - PPARG) was identified. In such cases, QOMIC is able to detect motif embeddings independently of existing regulatory interaction data. This demonstrates QOMIC's flexibility in identifying motif embeddings both with and without complete regulatory interactions.

SM 4: Running time performance of our algorithm

Next, we focus on QOMIC and the Ren et al method as they are the top two methods in terms of their success in finding motifs and examine their running times for network sizes from 200 to 1000 nodes. We omit the other two methods for brevity, as they find fewer motifs and the running time of Mfinder is substantially higher. Figure SM. 2 presents the results.

We observe that while the running time of the Ren et al method scales linearly along with the network sizes, the running time of QOMIC is independent with the network sizes. It comes from the fact that the running time of QOMIC is heavily depended on the complexity of the quantum circuit (i.e., the total number of quantum gates to encode the objective function f, and the actual time to execute these gates), and the efficiency of the optimizer in finding the optimal parameters. These factors do not strictly scale with the number of network nodes. Furthermore, as the network size grows, the running time of QOMIC gets closer and even potentially surpasses the running time of the Ren et al method. Specifically, for graphs with 1000 nodes, the time difference between QOMIC and that method are approximately 20 seconds in for the bifan pattern, and 2 seconds for the biparallel pattern. It is important to note that the quantum computing is still an evolving field. With the rapid development in the quantum computing technology, the running time of quantum computing can be further reduced. Thus, quantum computing is promising in solving complex biological problems efficiently.

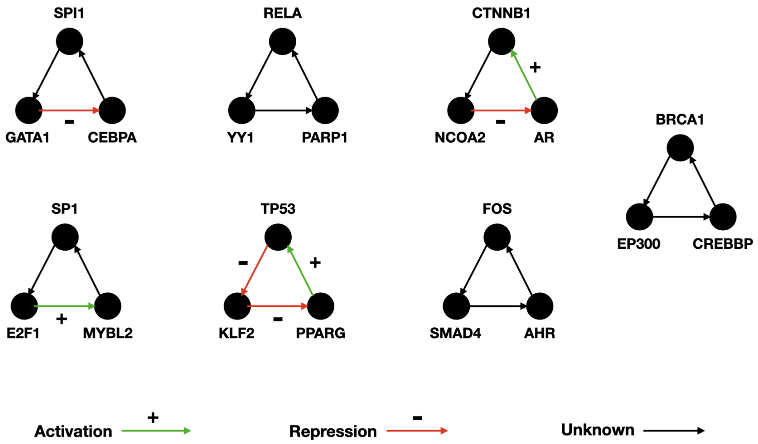

Fig. SM. 1. Cascade embeddings found by QOMIC in Alzionheimer's regulatory network. +/- signs indicate activation/repression relations. The edges without a sign indicate the interactions with unknown relation status.

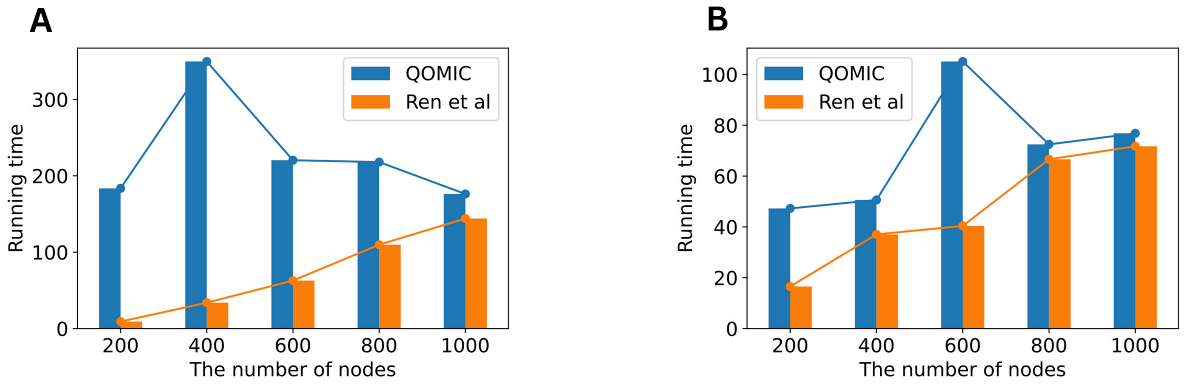

Fig. SM. 2. Analysis of the QOMIC and the Ren et al method in term of the running time. The analysis is illustrated in two motif types including (A) Bifan and (B) Biparallel.
